# The impact of guidelines on sterility precautions during indwelling urethral catheterization at two acute-care hospitals in Sweden - a descriptive survey

**DOI:** 10.1186/s12912-021-00619-x

**Published:** 2021-06-15

**Authors:** Aysel Kulbay, Eva Joelsson-Alm, Ann Tammelin

**Affiliations:** 1 Department of Medicine Solna, Karolinska Institutet, Unit of Infectious diseases, Solna, Stockholm, Sweden; 2 Department of Clinical Science and Education, Karolinska Institutet, Unit of Anaesthesiology and Intensive Care, Södersjukhuset, Stockholm, Sweden

**Keywords:** Urethral catheterization, Insertion technique, Guidelines, Compliance

## Abstract

**Background:**

To support a uniform and evidence-based practice for indwelling urinary catheterization in adults The European association of Urology Nurses (EAUN) published guidelines for this procedure in 2012. The Swedish national guidelines are based on the sterility precautions advocated by EAUN. Some hospitals have local guidelines with other requirements concerning sterility and leave to staff to decide how to perform the catheterization. The aim of this descriptive survey was to investigate the nurses´ self-reported sterility precautions during indwelling urethral catheterization at two acute-care hospitals, where the local guidelines differ in their sterility requirements. The study also aimed to analyze factors affecting conformity with sterility precautions in the EAUN-guidelines.

**Methods:**

A structured questionnaire with questions concerning the participant, working conditions and performance of indwelling urethral catheterization was left to 931 nurses in two acute care hospitals. Chi-square test, Fisher’s exact test and Mann-Whitney U-test were used for descriptive statistics. Logistic regression was used to analyze variables associated with practicing the sterility precautions in the EAUN-guidelines.

**Results:**

Answers were obtained from 852 persons (91.5%). Most of the participants called their insertion technique “non-sterile”. Regardless of designation of the technique the participants said that the indwelling urinary catheter (IUC) should be kept sterile during procedure. Despite that not everyone used sterile equipment to maintain sterility of the catheter. The nurses´ conformity with all the sterility precautions in the EAUN-guidelines were associated with working at departments for surgery and cardiology (OR 2.35, 95% CI 1.69–3.27), use of sterile set for catheterization (OR 2.06, 95% CI 1.42–2.97), use of sterile drapes for dressing on insertion area (OR 1.91, 95% CI 1.24–2.96) and using the term “sterile technique” for indwelling urethral catheterization (OR 1.64, 95% CI 1.11–2.43).

**Conclusions:**

Only 55–74% of the nurses practiced one or more precautions that secured sterility of the IUC thus demonstrating a gap between the EAUN-guidelines and the actual performance. Adherence to the guidelines was associated with factors that facilitated an aseptic performance such as using a sterile set and sterile drapes.

Healthcare-settings should ensure education and skill training including measures to ensure that the IUC is kept sterile during insertion.

## Background

Healthcare-associated urinary tract infection (HAUTI) is one of the most common healthcare-associated infections and is mostly linked to presence of an indwelling urinary catheter (IUC) [1–4]. In two point prevalence surveys of healthcare-associated infections (HAI) conducted by the European Centre for Disease Prevention and Control (ECDC) among in-patients at acute-care hospitals in European countries during 2011–2012 (1149 hospitals) and 2016–2017 (1209 hospitals), HAUTI constituted 19.0% and 18.9% of all HAI. In the first survey HAUTI was the third most common type of HAI and in the later survey the second most common [5, 6].

In a point prevalence survey conducted in 2018 among 3547 patients in acute-care hospitals in Stockholm, Sweden, the prevalence of HAUTI was 20.2% among inpatients with HAI [7].

Prevention of HAUTI has been the subject of many national guidelines in countries within and outside Europe [8–11]. With the believe that “excellent healthcare goes beyond geographical boundaries” ([12], p. 3) and to support a uniform evidence-based practice for indwelling urethral catheterization in adults the European association of Urology Nurses (EAUN) published guidelines for this procedure in 2012 [12].

One of the main strategies in preventing HAUTI in patients needing an IUC is to avoid contamination of the sterile IUC during insertion. This requires knowledge about sterility precautions and practice in aseptic technique during IUC-insertion on a regular basis [8–11]. To keep the IUC sterile during insertion EAUN recommends use of sterile lubricants, sterile equipment and aseptic technique [12]. The current Swedish national guidelines for indwelling urethral catheterization are based on the sterility precautions advocated by EAUN [13]. At the same time there exist local Swedish hospital guidelines with different requirements regarding sterility of the IUC and equipment during IUC-insertion. These local hospital guidelines supersede the national guidelines and vary with respect to how much they leave to staff to decide what equipment to use and how to perform the catheterization (see Table 1). A situation where international, national and local guidelines are available in parallel could easily cause confusion among staff performing urethral catheterization. Uncertainty might lead to inconsistent use and interpretation of terms, a non-uniform performance of the procedure and impaired patient safety. Therefore, we wanted to explore how the situation with several guidelines affected behavior in daily nursing and if this could jeopardize patient safety.

**Table 1:** Overview of guidelines concerning sterility precautions during indwelling urethral catheterization

Requirements	EAUN-guidelines [12]	Local guidelines Hospital A	Local guidelines Hospital B
Hand hygiene	Bactericidal alcohol hand rub	Bactericidal alcohol hand rub	Not mentioned
Sterility of the IUC during insertion	Sterile catheter	Sterile catheter	Non-sterile catheter
Preparation area for equipment	On a clean trolley	Not mentioned	Not mentioned
Insertion of the urethral catheter	With sterile gloves	With sterile gloves or sterile forceps held by non-sterile gloves	Not mentioned, refers to the national guidelines
Fluid for urinary bladder washouts	Sterile fluid	Sterile normal saline	Not mentioned

### Aim

The aim of the study was to investigate the nurses´ self-reported sterility precautions during indwelling urethral catheterization at two acute-care hospitals in Sweden, where the local guidelines differ in their sterility requirements. The study also aimed to analyze factors affecting the participants´ conformity with sterility precautions recommended in the EAUN-guidelines for indwelling urethral catheterization on adults.

## Methods

### Design and questionnaire

The study had a descriptive design and was based on a structured questionnaire, with 19 questions in total. Nine questions concerned the participant and the working conditions (profession, graduation year, years in profession, department, years at present department, ward, work shift, origin of the insertion technique, frequency of IUC-insertion) and ten questions concerned the indwelling urethral catheterization procedure (insertion technique, sterility of the IUC during insertion, hand hygiene prior to preparing for procedure, solution for periurethral cleaning, area for preparing equipment prior to IUC-insertion, sterility of the set for catheterization if used, sterility of dressing on insertion area if used, sterility of utilities used for IUC-insertion, disposable/reusable equipment used for IUC-insertion, fluid for urinary washouts if used).

The questionnaire was constructed by the researchers in collaboration with expertise in urology nursing and infection control and the questions concerning sterility precautions during IUC-insertion procedure were based on the EAUN-guidelines for indwelling urethral catheterization in adults [12]. The questionnaire was pilot tested for comprehensiveness on healthcare-personnel in urology at another hospital in Sweden prior to the study. Each person taking part in the pilot test was instructed to read the questions and describe thoughts and associations for each question and the corresponding answer options by thinking aloud. After completed questionnaire each person was interviewed about the need of any additional questions to describe the IUC-insertion procedure. The pilot test did not result in any changes of the questions or answer options.

### Setting

The study was conducted at two acute-care hospitals, hospital A and hospital B, with approximately 600 and 500 beds respectively, both situated in Stockholm County, Sweden. Wards for in-patient care at the departments for general surgery, cardiology and general internal medicine were chosen for the study as they had a similar level of care and urethral catheterization was performed regularly in all those wards by registered nurses and assistant nurses. At the time for the survey each hospital had local guidelines for indwelling urethral catheterization. The local guidelines at hospital A were launched in 2006 and updated in 2011. The local guidelines at hospital B were launched in 2011 and updated in 2013. See Table 1 for an overview of the required sterility precautions for indwelling urethral catheterization according to the EAUN-guidelines and both local hospital guidelines. EAUN used the term “sterile procedure” to summarize their requirements whereas hospital A used the heading “sterile technique” for their procedure and hospital B called their procedure “non-sterile technique”. At both hospitals it was possible for the newly employed nurses from the included wards to practice their IUC-insertion procedure at the hospital clinical training centers, but none of the wards required repeated training to keep up skills.

### Participants

Head Nurses at all 28 wards for in-patient care at departments for surgery, cardiology and internal medicine at both study hospitals were asked about taking part in the study after verbal and written information. At hospital A fourteen of 15 eligible wards (3 wards at the department for surgery, 4 wards at the department for cardiology and 7 wards at the department for internal medicine) accepted participation in the study. One Head Nurse (a ward at the department for surgery) declined study participation due to other ongoing studies and workload at the ward. At hospital B, all 13 eligible wards (3 wards at the department for surgery, 3 wards at the department for cardiology and 7 wards at the department for internal medicine) accepted participation. The participants were registered nurses and assistant nurses. Employees on sick leave, parental leave and temporary staff were not included.

### Data collection

**Fig. 1:**
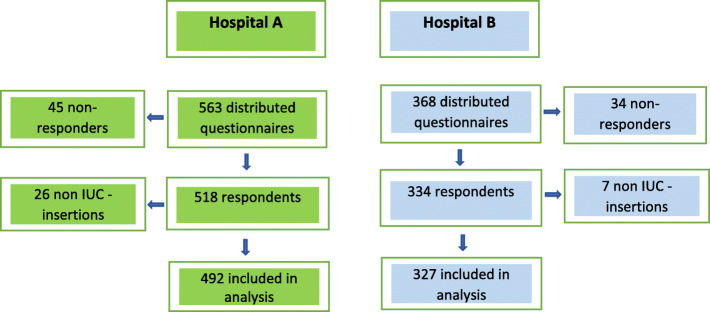
Flowchart of study inclusion process at both hospitals

Verbal and written information was given by the study conductor to the nurses and assistant nurses at the participating wards during ward meetings. The voluntary participation in the study was emphasized and the printed questionnaires were distributed by the study conductor or the Head Nurse to the employees fulfilling the inclusion criteria. The participants were instructed not to discuss the questions in the questionnaire during the study period. The Head Nurses reminded the staff about the questionnaire at ward meetings. Consent was implied when nurses voluntarily returned the answered questionnaires to the study conductor in preaddressed and sealed envelopes within 2 weeks after distribution. At hospital A, 563 questionnaires were distributed during December 2015–March 2016. At hospital B, 368 questionnaires were distributed during May 2016–January 2017. The answers in the returned questionnaires were anonymized prior to data analysis. See Fig. 1 for flowchart of the study inclusion process.

### Data analysis

Differences in background characteristics and indwelling urethral catheterization procedure of the participants were evaluated with Chi-square test, Fisher’s exact test and Mann-Whitney U-test. Continuous variables were expressed as medians (IQR) and categorical variables as numbers (%).

Binary logistic regression was used to identify variables associated with practicing the sterility precautions for indwelling urethral catheterization required in the EAUN-guidelines. The dependent variable was performing in agreement with all the five components of the sterility precautions in the EAUN-guidelines as described in Table 1 (bactericidal alcohol hand rub, sterile catheter, on a clean trolley, with sterile gloves, sterile fluid). Thirteen explanatory variables were tested, among these eight background factors (hospital, department, profession, years in profession, years at present department, work shift, frequency of IUC-insertion, origin of the insertion-technique) and five technique-related factors (type of insertion-technique, periurethral cleansing solution, use of set for catheterization, dressing on insertion area and disposable vs reusable equipment) which could affect the sterility during IUC-insertion.

We categorized the following variables into two groups: department (internal medicine and cardiology/surgery), years in profession (0–2 years and > 2 years), work shift (day/evening/alternating shift and night shift), frequency of IUC-insertion (each week or month and less than each month), origin of the insertion technique (according to the hospital guidelines and other answers), insertion technique (sterile technique and non-sterile technique), use of set for catheterization (sterile set and non-sterile set/no set used), dressing on insertion area (sterile drapes and non-sterile drapes/no drapes) and equipment for catheterization (disposable and reusable/don’t know). Periurethral cleansing solution was categorized into three groups (soap/tap water, sterile normal saline and other solutions) to reflect to participants´ choices for periurethral cleaning prior to IUC-insertion. First, univariable analyses was used to study crude associations of each explanatory variable with the odds (OR) of factors affecting the participants´ conformity with the sterility precautions required in the EAUN-guidelines. Secondly, multivariable logistic models were used in a backward and forward procedure to study the adjusted associations. Variables with a *p*-value < 0.10 in the univariable analyses were included in the multivariable analyses. The associations are presented as odds ratios (OR) with 95% confidence intervals (CI). Finally, Hosmer-Lemeshow goodness-of-fit test was used to assess the adjusted model, with a p-level above 0.05 indicating an acceptable fit. The IBM Statistical Package for the Social Sciences (SPSS) version 26.0 (IBM Corp., Armonk, NY, USA) was used for all analyses and a two-sided *p*-value of < 0.05 was considered statistically significant.

## Results

### Participants

Answers were obtained from 518 of 563 included persons (92%) at hospital A and from 334 of 368 included persons (91%) at hospital B. Among the respondents at both hospitals 33 of them (26 at hospital A, 7 at hospital B) answered that they never inserted indwelling urinary catheters and were excluded from analysis. Not all questions were answered by every participant, thus leading to different numbers of analyzed answers for each question.

In total, answers from 492 participants at hospital A and 327 answers from participants at hospital B were analyzed.

The characteristics of the participants from both study hospitals are displayed in Table 2.

**Table 2:** Characteristics of participants from the two study hospitals

Characteristics	Hospital A (*n* = 492)	Hospital B (*n* = 327)	*p*-value
Profession, n (%)			0.658
Registered nurse	311 (63.2)	201 (61.5)	
Assistant nurse	181 (36.8)	126 (38.5)	
Departments, n (%)			< 0.001*
Cardiology	186 (37.8)	62 (19.0)	
Internal Medicine	223 (45.3)	180 (55.0)	
Surgery	83 (16.9)	85 (26.0)	
Years in profession, median (IQR)	8 (3.5–18)	5 (2–13)	< 0.001*
Graduation year			0.045*
Before 1990	65 (13.3)	25 (7.7)	
1990–1999	80 (16.4)	48 (14.8)	
After 1999	342 (70.1)	252 (77.5)	
Missing	1 (0.2)	0 (0.0)	
Years at present department, median (IQR)	3 (1–8)	2 (1–6)	0.019*
Work shift, n (%)			< 0.001*
Day/evening shift	237 (48.2)	198 (60.6)	
Night shift	66 (13.4)	51 (15.6)	
Alternating shifts (day/evening/night)	181 (36.8)	77 (23.5)	
Other answers	8 (1.6)	1 (0.3)	
Frequency of IUC-insertion, n (%)			0.657
2 times or more/week	26 (5.3)	20 (6.1)	
1–5 times/month	212 (43.1)	143 (43.7)	
Less frequently	249 (50.6)	163 (49.8)	
Other answers	2 (0.4)	0	
Missing	3 (0.6)	1 (0.3)	
Origin of the insertion technique, n (%)			0.459
The hospital guidelines	287 (60.3)	202 (62.9)	
Other answers	189 (39.7)	119 (37.1)	
(national guideline, nursing school, local routine at ward, don’t know)			

* A *p*-value < 0.05 was considered as a statistically significant difference between hospital A and hospital B

The participation is described on department level hence ward is not included in Table 2.

At hospital A, a higher proportion of participants worked at the department of cardiology, worked alternating shifts (both day, evening and night shifts) and had longer professional experience compared to the participants at hospital B.

### Sterility precautions during procedure

The participants’ answers about their denomination of the insertion technique – sterile or non-sterile – and sterility precautions during indwelling urethral catheterization are presented in Table 3.

**Table 3:** Participants´ answers about sterility precautions during indwelling urethral catheterization

Survey questions	Both hospitals	Hospital A	Hospital B	*p*-value*
Insertion technique, n (%)				0.011**
Sterile technique***	206 (25.3)	**139** (28.5)	67 (20.5)	
Non-sterile technique	608 (74.7)	348 (71.5)	**260** (79.5)	
Sterility of the IUC during insertion, n (%)				0.175
Sterile IUC***	658 (80.6)	**403** (82.2)	255 (78.2)	
Non-sterile IUC	158 (19.4)	87 (17.8)	**71** (21.8)	
Hand hygiene prior to preparing for procedure, n (%)				0.079
Disinfected hands***	730 (89.6)	**446** (91.2)	284 (87.1)	
Other answers (e.g. clean hands)	85 (10.4)	43 (8.8)	42 (12.9)	
Solution for periurethral cleaning, n (%)				0.640
*S*oap and tap water	678 (83.3)	**404** (82.8)	274 (84.0)	
Sterile normal saline 9 mg/ml	75 (9.2)	44 (9.0)	31 (9.5)	
Other answers	61 (7.5)	40 (8.2)	21 (6.4)	
Area for preparing equipment prior to IUC-insertion, n (%)				< 0.001**
On a disinfected trolley***	545 (66.7)	304 (55.8)	241 (74.2)	
Other answers (e.g. bedside table, bed)	272 (33.3)	188 (38.2)	84 (25.8)	
Set for catheterization, n (%)				0.127
Sterile set	460 (57.0)	274 (56.7)	186 (57.4)	
Non-sterile set	206 (25.5)	115 (23.8)	91 (28.1)	
Do not use a set	141 (17.5)	94 (19.5)	47 (14.5)	
Dressing on insertion area, n (%)				0.225
Sterile drapes for dressing	148 (18.2)	95 (19.5)	53 (16.2)	
Non-sterile drapes for dressing	568 (69.7)	329 (67.4)	239 (73.1)	
No drapes used on insertion area	99 (12.1)	64 (13.1)	35 (10.7)	
Insertion of the IUC, n (%)				0.042**
With sterile gloves/forceps/	537 (65.8)	336 (68.6)	201 (61.7)	
Inner cover*** With non-sterile gloves/forceps	279 (34.2)	154 (31.4)	125 (38.3)	
Type of equipment for IUC-insertion, n (%)				0.004**
Disposable equipment	774 (95.3)	472 (97.1)	302 (92.6)	
Reusable equipment	38 (4.7)	14 (2.9)	24 (7.4)	
Fluid for urinary bladder washouts, n (%)				0.331
Sterile normal saline 9 mg/ml***	713 (97.1)	434 (97.3)	279 (96.9)	
Tap water	8 (1.1)	3 (0.7)	5 (1.7)	
Other fluids (e.g. disinfectants)	13 (1.8)	9 (2.0)	4 (1.4)	

Bold figure indicates that the hospital guidelines were followed at each hospital.

* Comparison between hospital A and hospital B

** A *p*-value < 0.05 was considered as a statistically significant difference between Hospital A and Hospital B

*** Correct aseptic technique according to EAUN-guidelines

Most of the nurses from both study hospitals called their insertion technique “non-sterile” (hospital A 71.5%, hospital B 79.5%), the nurses from hospital A however reported this significantly less often compared to nurses from hospital B (*p*-value 0.011).

Although a majority of the nurses at both study hospitals answered that the IUC should be kept sterile during insertion (hospital A 82.2%, hospital B 78.2%), nurses at hospital A more often inserted the IUC with sterile gloves or a sterile forceps or by holding the catheter’s inner plastic cover (hospital A 68.6% vs hospital B 61.7%, *p*-value 0.042). During the procedure use of disposable equipment was more common at hospital A (hospital A 97.1% vs hospital B 92.6%, *p-*value 0.004).

Irrespective of insertion technique a sterile set for catheterization was used by over half of the participating nurses from both study hospitals. Only 16–20% of nurses claimed using sterile drapes for dressing on the insertion area in efforts to create a protective field and prevent contamination of the IUC during insertion. Dressing on insertion area was not required in neither of the local hospital guidelines.

### Performing in agreement with sterility precautions in the EAUN-guidelines

The univariable analysis identified four explanatory variables that were significantly associated with performing in agreement with the five components of the sterility precautions in the EAUN-guidelines (see Table 1). After performing a multivariable logistic regression analysis to study the adjusted associations for those four variables the significant associations remained. The four variables were: working at department for surgery and cardiology (OR 2.35, 95% CI 1.69–3.27), use of sterile set for catheterization (OR 2.06, 95% CI 1.42–2.97), use of sterile drapes for dressing on insertion area during procedure (OR 1.91, 95% CI 1.24–2.96) and using the term “sterile technique” for indwelling urethral catheterization (OR 1.64, 95% CI 1.11–2.43).

See Table 4 for details in the univariable and multivariable analyses.

**Table 4:** Factors associated with performing indwelling catheterization in agreement with the sterility precautions in the EAUN-guidelines*

Explanatory variable	Univariable OR (95% CI) *p*-value		Multivariable OR (95% CI) *p-*value	
Hospital		0.930	N/A	
Hospital A	0.99 (0.72–1.34)			
Hospital B	Reference			
Department		< 0.001**		< 0.001***
Cardiology & Surgery	2.31 (1.70–3.16)		(1.69–3.27)	
Internal medicine	Reference		Reference	
Profession		0.653		
Registered nurse	Reference		N/A	
Assistant nurse	1.07 (0.79–1.47)			
Years in profession		0.109		
0–2 years	1.35 (0.94–1.94)		N/A	
> 2 years	Reference			
Years at present department		0.086**		0.230
0–2 years	1.31 (0.96–1.77)		1.22 (0.88–1.70)	
> 2 years	Reference		Reference	
Work shift		0.49		
Day/evening/alternating shift	1.25 (0.79–1.95)		N/A	
Night shift	Reference			
Frequency of IUC-insertion		0.742		
Each week or month	1.05 (0.78–1.42)		N/A	
Less than each month	Reference			
Origin of the insertion technique		0.654		
According to the hospital guidelines	1.07 (0.79–1.47)		N/A	
Other answers	Reference			
Insertion technique		< 0.001**		0.014***
Sterile technique	2.61 (1.86–3.66)		1.64 (1.11–2.43)	
Non-sterile technique	Reference		Reference	
Periurethral cleansing solution		0.127	N/A	
Soap and tap water	0.80 (0.45–1.41)			
Sterile normal saline	1.33 (0.64–2.74)			
Other solutions	Reference			
Use of set for catheterization		< 0.001**		< 0.001***
Sterile set	2.84 (2.04–3.94)		2.06 (1.42–2.97)	
Non-sterile set or no set used	Reference		Reference	
Dressing on insertion area		< 0.001**		0.004***
Sterile drapes	3.17 (2.16–4.65)		1.91 (1.24–2.96)	
Non-sterile drapes or no drapes	Reference		Reference	
Equipment for catheterization		0.123		
Disposable	Reference		N/A	
Reusable or don’t know	1.71 (0.86–3.37)			

*The five components of the sterility precautions in the EAUN-guidelines are bactericidal alcohol hand rub, sterile catheter, on a clean trolley, insertion with sterile gloves and sterile fluid for bladder washouts

** Variables with a *p-*value < 0.1 in the univariable analyses were included in the multivariable analyses

*** Variables with a *p*-value < 0.05 in the multivariable analyses were considered as statistically significant associations

*N/A* not applicable.

## Discussion

### Sterility precautions

Maintaining the sterility of the IUC during insertion is one of the cornerstones in evidence-based international guidelines for prevention of HAUTI among patients in need of an IUC. The sterility of the catheter is kept by using sterile equipment, lubricants and solutions, by proper hand hygiene and by skills in ensuring not to contaminate the IUC during the whole procedure [8–12]. In this study it was mostly considered by the participants that the IUC should be kept sterile during insertion (hospital A 82.2%, hospital B 78.2%) which is in accordance with the EAUN-guidelines but not required by the local guidelines at hospital B. Despite of that only 62–69% of the participants used sterile gloves/forceps for catheter insertion or practiced a non-touch technique by keeping the catheter sterile within its inner plastic cover during insertion. The nurses at hospital A reported higher adherence to those techniques compared to hospital B (*p*-value 0.04).

Sterile drapes on the insertion area could be used to protect a catheter from unintended contact with the patient’s legs or bed linen. This is not required in the EAUN-guidelines but is advocated in national guidelines from for example the United States of America and Ireland [8, 10]. Only 16–20% of the participants at both study hospitals used sterile drapes on the insertion area to protect the sterile catheter from contamination during catheterization.

Another measure aimed at securing an aseptic procedure is to use a disinfected trolley to prepare the equipment needed for catheterization. This arrangement was reported from 56 to 74% of the participants and significantly more often at hospital B (*p*-value < 0.001).

A standardized set for urethral catheterization, including all necessary sterile equipment, such as gloves, forceps, fenestrated drapes, gallipots and swabs can both facilitate a uniform behavior when performing indwelling urethral catheterization and secure sterility of the IUC throughout the whole procedure. A pre-prepared set ensures that no necessary item is forgotten. The facilitating effect of a pre-prepared set for aseptic IUC-insertion is also reported in a study by Mizerek et al. [14]. In our study almost 83% of the participants used a pre-prepared set for catheterization though only 57% used a sterile set.

### Sterile or non-sterile technique

Although 80% of participants advocated the maintenance of IUC sterility during insertion and ~ 60% reported practicing that behavior, most of the study participants at both hospitals called their insertion technique “non-sterile” (hospital A 71.5%, hospital B 79.5%). The nurses from hospital A had significantly lower reports of this compared to nurses from hospital B (*p*-value 0.011). This might be associated with the requirement for keeping the catheter sterile and mentioning the term “sterile technique” in the local hospital guidelines at hospital A contrary to the local guidelines at hospital B where “non-sterile technique” was advocated.

The use of the term “non-sterile technique” for the procedure irrespective of how it actually was performed might originate from the introduction of “non-sterile technique” in the Swedish national guidelines for indwelling urethral catheterization during the 1990s. Those guidelines were influenced by a small randomized clinical trial conducted by Carapeti et al. in 1994. In this study the authors compared “sterile technique” with “non-sterile technique” for short-term indwelling urethral catheterization on patients undergoing elective general surgery [15]. The study found no statistically significant difference in UTI incidence between the compared insertion techniques, (11% with “non-sterile technique”, 9.5% with “sterile technique”, *p*-value > 0.1) and the authors recommended the cheaper “non-sterile technique”. Important to notice is however, that the IUC was kept sterile during both procedures by holding the sterile IUC within its inner plastic cover during insertion. “Non-sterile technique” was introduced in the revised Swedish national guidelines launched in 1994 and included use of soap and tap water for periurethral cleaning, no dressing on insertion area, use of non-sterile equipment and non-sterile gloves when inserting the IUC [16]. Unfortunately, the emphasis on not touching the sterile IUC was left out. This opinion of the proper way to perform indwelling urethral catheterization has dominated in Sweden and thus influenced what nurses have been taught during education and professional work until an updated version of the national guidelines based on same sterility precautions as in the EAUN-guidelines was launched in 2015. This return to an earlier approach to the principles of keeping the sterility of the IUC during insertion in the national guidelines does not seem to have had an impact on what the participants in this study called their insertion technique.

A variety of terms for IUC-insertion techniques are found in scientific and educational literature. Further, healthcare settings can have different interpretations of a specific insertion technique. For example, EAUN stresses that “non-sterile technique” is applicable only for a patient performing intermittent self-catheterization at home [17]. The inconsistent use of different terms for insertion technique during urethral catheterization and uncertainties in understanding how proper aseptic insertion of the sterile catheter is accomplished by nurses has also been reported by others [18–21].

National and local hospital guidelines for indwelling urethral catheterization should use a harmonized description of the term “sterile technique” accompanied with an explanation of what necessary sterile equipment to use for successful aseptic procedure, with the emphasis on the use of a sterile set for catheterization and sterile drapes on the insertion area to create a protective field for the sterile urinary catheter so that it is not accidentally contaminated during procedure.

### Conformity with the EAUN-guidelines

The different requirements for keeping the catheter sterile in the local hospital guidelines from hospital A and hospital B did not affect the adherence to the EAUN-guidelines (OR 0.99, 95% CI 0.72–1.34).

Performing indwelling urethral catheterization according to the EAUN-guidelines was associated with working at departments for surgery and cardiology (*p*-value < 0.001). An explanation may be that skill training was more common at those departments compared with the department of internal medicine.

An association with performing according to the EAUN-guidelines was also found for the use of sterile set for catheterization (*p*-value < 0.001) and sterile drapes for dressing on insertion area during procedure (*p*-value 0.004). There was also an association between adherence to the EAUN-guidelines and using the term “sterile technique” for indwelling urethral catheterization (*p*-value 0.014). A possible explanation for this may be that the term “sterile” is easier to relate to an aseptic performance than the term “non-sterile”.

On an overall level we believe that the association between those variables and performing according to the EAUN-guidelines demonstrates the knowledge and understanding of aseptic technique by the individual nurse. This knowledge could have been achieved during education or employment and is obviously more influential for the actual behavior than a written hospital guideline.

We have chosen to analyze association of the explanatory variables and performing in accordance with all the five components of sterility precautions in the EAUN-guidelines as we believe that the five components act like a bundle. When urinary catheterization is performed all the five components should be kept to.

### Problems and potential interventions

Different requirements on sterility and equipment in the local hospital guidelines, combined with the lack of a detailed description of the IUC-insertion process are factors that counteract a uniform performance of indwelling urethral catheterization. This may jeopardize the patient safety.

In addition to harmonized guidelines the need for training in aseptic preparation of sterile equipment is stressed by the EAUN-guidelines and other international guidelines [8–12]. Lo et al. states that only nurses passing competence assessment in aseptic IUC-insertion should perform indwelling urethral catheterization and the nurses´ competencies should be reassessed on a regular basis [22]. According to Walsh et al. “computer-assisted learning” is as effective as “expert-assisted learning” for basic knowledge and skills training in aseptic IUC-insertion [23]. In an experimental study conducted by Todsen et al. they found that the obtained skills in aseptic IUC-insertion during a theoretical course combined with a practical training on mannequins were still retained when the participants´ performances were reassessed 6 weeks later when performing IUC-insertion on real patients in a clinical setting [24].

Furthermore, in a multi-modal study conducted by Ara et al. with several activities including for example theoretical education and practical training in standard precautions and aseptic technique, visual reminders, monitoring and feedback on a regular basis resulted in a significant overall improvement of aseptic maintenance of sterile equipment [25]. This multi-modal intervention approach for prevention of HAI is also advocated by the World Health Organization (WHO) in Guidelines on Core Components of Infection Prevention and Control Programmes at the national and acute health care facility level [26].

The results from our study support the need for the healthcare-settings to have a strategy regarding repeated training of the staff in aseptic IUC-insertion procedure and how to implement changes in updated guidelines for urethral catheterization.

### Methodological considerations

A limitation of our study is that it is based on self-reported description of practice and not observation of the actual performance during indwelling urethral catheterization. Using a questionnaire, however, made it possible to cost-efficiently reach many more nurses from different departments at two hospitals, with different sterility requirements in local hospital guidelines, than observation would have done. A validation of the procedure described by the participants requires an observational study of the practiced skills such as conducted by Manojlovich et al. [21]. Another limitation may be that the study did not include physicians. Although IUC-insertion can be performed by physicians, urethral catheterization in Sweden is performed mostly by nurses hence the focus on nurses in the study. The guidelines for catheterization are usually written by registered nurses.

## Conclusion

There is a gap between the description of sterility precautions in the national guidelines for urethral catheterization based on evidence-based guidelines from EAUN and the nurses´ self-reported sterility precautions during procedure. For a uniform performance securing sterility of the urinary catheter, and thus patient safety, updated guidelines should include a clear description of what sterile equipment to use, how and where to prepare for procedure and how to maintain the sterility of the IUC during procedure [18]. As there is an obvious confusion about the meaning of the term “non-sterile technique” this should be omitted in any guideline. Healthcare-settings should ensure educational support and skill training for nurses including insertion of the sterile IUC with a sterile forceps or sterile gloves. Using sterile sets for catheterization and drapes on insertion area should also be taught.

## Data Availability

The datasets used and analyzed during the current study are available from the corresponding author on reasonable request.

## Abbreviations

HAUTI: Healthcare-associated urinary tract infection
IUC: Indwelling urinary catheter
ECDC: The European Centre for Disease Prevention and Control
EAUN: The European Association of Urology Nurses
IQR: Interquartile range
OR: Odds ratio
CI: Confidence interval
SPSS: The IBM Statistical Package for the Social Sciences
WHO: World Health Organization
